# Antitumor Effects of a Sirtuin Inhibitor, Tenovin-6, against Gastric Cancer Cells via Death Receptor 5 Up-Regulation

**DOI:** 10.1371/journal.pone.0102831

**Published:** 2014-07-17

**Authors:** Sachiko Hirai, Shinji Endo, Rie Saito, Mitsuaki Hirose, Takunori Ueno, Hideo Suzuki, Kenji Yamato, Masato Abei, Ichinosuke Hyodo

**Affiliations:** Department of Gastroenterology, Institute of Clinical Medicine, Graduate School of Comprehensive Human Sciences, University of Tsukuba, Tsukuba, Ibaraki, Japan; University of Illinois at Chicago, United States of America

## Abstract

Up-regulated sirtuin 1 (SIRT1), an NAD^+^-dependent class III histone deacetylase, deacetylates p53 and inhibits its transcriptional activity, leading to cell survival. SIRT1 overexpression has been reported to predict poor survival in some malignancies, including gastric cancer. However, the antitumor effect of SIRT1 inhibition remains elusive in gastric cancer. Here, we investigated the antitumor mechanisms of a sirtuin inhibitor, tenovin-6, in seven human gastric cancer cell lines (four cell lines with wild-type *TP53*, two with mutant-type *TP53*, and one with null *TP53*). Interestingly, tenovin-6 induced apoptosis in all cell lines, not only those with wild-type *TP53,* but also mutant-type and null versions, accompanied by up-regulation of death receptor 5 (DR5). In the KatoIII cell line (*TP53*-null), DR5 silencing markedly attenuated tenovin-6-induced apoptosis, suggesting that the pivotal mechanism behind its antitumor effects is based on activation of the death receptor signal pathway. Although endoplasmic reticulum stress caused by sirtuin inhibitors was reported to induce DR5 up-regulation in other cancer cell lines, we could not find marked activation of its related molecules, such as ATF6, PERK, and CHOP, in gastric cancer cells treated with tenovin-6. Tenovin-6 in combination with docetaxel or SN-38 exerted a slight to moderate synergistic cytotoxicity against gastric cancer cells. In conclusion, tenovin-6 has potent antitumor activity against human gastric cancer cells via DR5 up-regulation. Our results should be helpful for the future clinical development of sirtuin inhibitors.

## Introduction

Gastric cancer is one of the major causes of cancer death around the world [1], [2]. Although various chemotherapies for advanced gastric cancer have been developed, the prognosis is still poor and novel anticancer drugs for gastric cancer are needed. Gastric cancer is a biologically and genetically heterogeneous cancer involving numerous genetic mutations and epigenetic alternations [3]. Among these, abnormalities of the *TP53* tumor suppressor gene play an important role in tumorigenesis [4], [5]. Approximately 30% of patients with gastric cancer have *TP53* mutation [6]. Even in cancer cells with wild-type (wt) *TP53*, it has been reported that the function of *TP53* is suppressed by negative regulation including ubiquitination, methylation, and deacetylation [7], [8]. In this context, it would be a promising strategy to assume that inhibition of these negative regulators results in enhancement of antitumor effects through activation of p53 in wt *TP53* cancers. Murine double minute 2 (MDM2) is a major physiological antagonist of p53 [7]. We previously reported that an MDM2 inhibitor, nutlin-3, demonstrated potent antitumor effects against gastric cancer cells through activation of the p53 pathway [9].

Sirtuin 1 (SIRT1), an NAD^+^-dependent histone deacetylase (HDAC), has a variety of functions involved in chromatin silencing, longevity, and genomic stability. It is found in the nucleus and acts as a sensor of cell metabolic status in survival and senescence under genotoxic and oxidative stress [10], [11]. Besides histone deacetylation, these functions partly depend on the deacetylation of various non-histone proteins that include transcriptional factors: p53, forkhead box (FOXO) family proteins, nuclear factor κB, c-MYC, N-MYC, E2F1, and hypoxia-inducible transcription factors (HIF) 1α/2α; chromatin-related enzymes: histone acetyltransferase, p300, DNA-dependent kinase subunit Ku80, and TIP60; DNA repair elements: Ku70, RAD51, and NBS1; and cell-signaling factors: STAT3, β-catenin, and Smad7 [11]–[13]. SIRT1 physiologically interacts with p53 and attenuates its functions through deacetylation at its C-terminal Lys382 residue [12]. Overexpression of SIRT1 was found in many cancers, such as stomach and colon [10], [14], and reported to function as a tumor promoter. SIRT2 is one of the cytoplasmic NAD^+^-dependent histone deacetylases and deacetylates histone H3 lysine 56 (H3K56) and α-tubulin. It also shares non-histone substrates of FOXO1, FOXO3, and p53 with SIRT1 [11]. However, the exact role of SIRT2 remains elusive in cancer biology.

Against this background, we investigated whether tenovin-6, a small-molecule compound that inhibits SIRT1 and SIRT2 functions [15], [16], exerted antitumor effects through activation of the p53 pathway in gastric cancer cells. Recently, it has been reported that SIRT inhibitors up-regulated the death receptor 5 (DR5), a member of the tumor necrosis factor receptor family, in some cancers [17], [18]. We additionally studied the involvement of this receptor in the antitumor activity of tenovin-6 for gastric cancer. Furthermore, we examined the synergism of tenovin-6 with conventional cytotoxic drugs for the future clinical development in gastric cancer.

## Materials and Methods

### Cell lines

Seven gastric cancer cell lines were used: four cell lines with wt *TP53* (MKN-45, NUGC-4, STKM-2, SNU-1), two cell lines with mutant-type (mt) *TP53* (NUGC-3, STKM-1), and one cell line with null *TP53* (KatoIII) [19]–[21]. Cell lines with wt *TP53* (MCF-7 breast cancer, HEK293 human embryonic kidney cells) and MRC-5 normal human fibroblasts were included as controls in this study. MKN-45, NUGC-4, KatoIII, and MRC-5 cell lines were obtained from RIKEN BRC Cell Bank (Tsukuba, Japan). SNU-1 and MCF-7 cell lines were purchased from the American Type Culture Collection (Rockville, MD). NUGC-3 and HEK293 cell lines were obtained from Health Science Research Resources Bank (Osaka, Japan). STKM-1 and STKM-2 cell lines were kindly provided by Dr. Shunsuke Yanoma (Yokohama City University, School of Medicine, Japan).

### Chemicals

Tenovin-6 was purchased from Cayman Chemical Company (Ann Arbor, MI). Docetaxel, SN-38, cisplatin, 5-fluorouracil (5-FU), doxorubicin and thapsigargin were obtained from Wako (Osaka, Japan). They were dissolved in dimethyl sulfoxide (DMSO) at a concentration of 20 mM and aliquots were stored at -20 °C. Stock solutions were diluted to the desired final concentrations with growth medium prior to use.

### Antibodies and Western blot analysis

SDS-polyaclylamidegel electrophoresis and Western blotting were performed as previously described [22]. The primary and secondary antibodies used were as follows. Rabbit polyclonal antibodies against SIRT1 (D739), acetylated (Ac)-p53 (Lys382), phosphorylated (Phospho)-p53 (Ser15), Bcl-2, Ac-α-tubulin (Lys40), death receptor 5 (DR5), Fas-associated death domain (FADD), cleaved poly(ADP)-ribose polymerase (PARP) (Asp214), and mouse monoclonal antibodies against p21*^Waf/Cip1^* (DCS60), histone H3 (96C10), β-actin (8H10D10), α -tubulin (DM1A) and C/EBP homologue protein (CHOP) (L63F7), and rabbit monoclonal antibodies against TRAIL (C92B9), caspase-3 (8G10), inositol-requiring enzyme (IRE) 1α (14C10), and phospho-RNA-dependent protein kinase-like endoplasmic reticulum kinase (PERK) (16F8) were obtained from Cell Signaling Technology (Danvers, MA). Mouse monoclonal antibody to p53 (BP53-12) was purchased from Cell Science (Canton, MA), anti-SIRT2 (4B11) monoclonal antibody was from Sigma-Aldrich. (St. Louis, MO), and anti-activating transcription factor 6 (ATF6) monoclonal antibody (70B1413.1) was from Enzo Life Science (Farmingdale, NY). Rabbit polyclonal antibody to Ac-histone H3 (Lys18) was from Merck Millipore (Billerica, MA). Both horseradish peroxidase (HRP)-conjugated anti-mouse IgG sheep and anti-rabbit IgG donkey sera were from GE Healthcare (Buckinghamshire, UK). Antibody binding was detected using an ECL Prime Western Blotting Detection System (GE Healthcare), in accordance with the manufacturer's protocol. The signal intensity was quantified using Ez-capture II chemiluminescence imaging system (Atto, Tokyo, Japan).

### Real-time quantitative PCR for analysis of *SIRT1* and *SIRT2* gene expression

RNA samples were extracted from cell lysate using a High Pure RNA Isolation kit (Roche Diagnostics, Mannheim, Germany), in accordance with the manufacturer's instructions. After the genomic DNA was removed by DNase, cDNA was prepared using a High Capacity RNA-to-cDNA kit (Life Technologies Corp., Carlsbad, CA). Real-time quantitative PCR was performed using an Applied Biosystems 7500 Fast Real-Time PCR System (Applied Biosystems, Foster City, CA). Primers and TaqMan probe for *SIRT1* and *SIRT2* were obtained from Applied Biosystems (Assay ID: Hs01009005 and Hs00247263, respectively), and those for *18S ribosomal RNA (18S rRNA)* designed and synthesized by Sigma-Aldrich were as follows: 5′-AACCCGTTGAACCCCATTCG (forward primer), 5′-CGGGCGGTGTGTACAAAGG (reverse primer), 5′-AACGCAAGCTTATGACCCGCACTTACTGG (probe). Reactions were performed in triplicate under standard thermocycling conditions using 30 ng of cDNA, 900 nM primers, 250 nM probes, and a Taqman Gene Expression Master Mix (Applied Biosystems), in accordance with the manufacturer's protocol.

RNAs extracted from cells were analyzed for the relative amounts of the target gene (*SIRT1, SIRT2*) and the reference gene (*18S rRNA*) by quantitative real-time PCR.

### WST-8 cell viability assays

WST-8 colorimetric assays were performed using a Cell Counting Kit-8 (Dojin Laboratories, Kumamoto, Japan), in accordance with the manufacturer's protocol. Cells were seeded into 96-well plates at a density of 5×10^3^ cells per well with 100 µl of culture medium for 24 h, treated with tenovin-6 for 72 h, incubated in the presence of WST-8, and then analyzed with an iMark microplate reader (Bio-Rad, Hercules, CA).

### Analysis of apoptosis by flow cytometry

Cells were seeded in 60-mm dishes at a density of 5×10^5^ per dish. After incubation with tenovin-6 (10 µM) or an equivalent amount of DMSO for 72 h, cells were gently lifted with Accutase (US Biotechnologies, Parker Ford, PA) at room temperature for 10 min. The cells were then washed once with phosphate buffered saline. Apoptotic cells were detected by double staining with propidium iodide (PI) and fluorescein isothiocyanate (FITC)-labeled annexin V using an Annexin V-FITC Apoptosis Detection Kit (Beckman Coulter, Brea, CA), in accordance with the manufacturer's protocol. Flow cytometric analysis was then performed with a FACS Calibur flow cytometer (BD Biosciences, Franklin Lakes, NJ) and CELLQuest software (BD Biosciences).

### siRNA targeting *DR5*

siRNA targeting DR5 was designed using siDirect software (http://sidirect2.rnai.jp/), as reported previously [22]. siRNA transfection was carried out using Lipofectamine RNAiMAX (Invitrogen, Carlsbad, CA), in accordance with the manufacturer's instructions. Control siRNA was an artificial sequence designed to have the least homology to human and mouse genes. The sense and antisense strands of siRNA used in this study were as follows: DR5, 5'-CCGUUUGUGCGUACUUUGAGA-3' (sense), 5'-UCAAAGUACGCACAAACGGAA-3' (antisense); control siRNA, 5'-CCGUACUAGCCAUUAUGCGUC-3' (sense), 5'-CGCAUAAUGGCUAGUACGGGU-3' (antisense).

For analysis of the effects of siRNA on cell growth and viability, cells were plated at a low density (1×10^3^ cells per well) in 96-well plates containing 100 µl of RPMI1640 medium with 10% fetal calf serum (Sigma-Aldrich). The viability of transfected cells was assessed 72 and 120 h after transfection by WST-8 assay.

### Combination index

To determine whether tenovin-6 can enhance the antitumor effects of conventional chemotherapeutic agents, we used a combination index (CI) and an isobologram calculated using CalcuSyn software (Cambridge, UK), in accordance with the Chou and Talalay median effect principle [23]. In this analysis, CI >1.3 indicates antagonism; CI  = 1.1–1.3 moderate antagonism; CI  = 0.9–1.1 additive effect; CI  = 0.8–0.9 slight synergism; CI  = 0.6–0.8 moderate synergism; CI  = 0.4–0.6 synergism; and CI  = 0.2–0.4 strong synergism.

### Statistical analysis

All experiments were performed in triplicate and were repeated at least three times. All data are expressed as mean ± standard deviation (SD). The significance of differences was determined by Student's t-test and Dunnett's test. *p*-values <0.05 were considered significant.

## Results

### Expression of SIRT1, SIRT2, and acetylated (Ac)-p53 in gastric cancer cell lines

**Figure 1:**
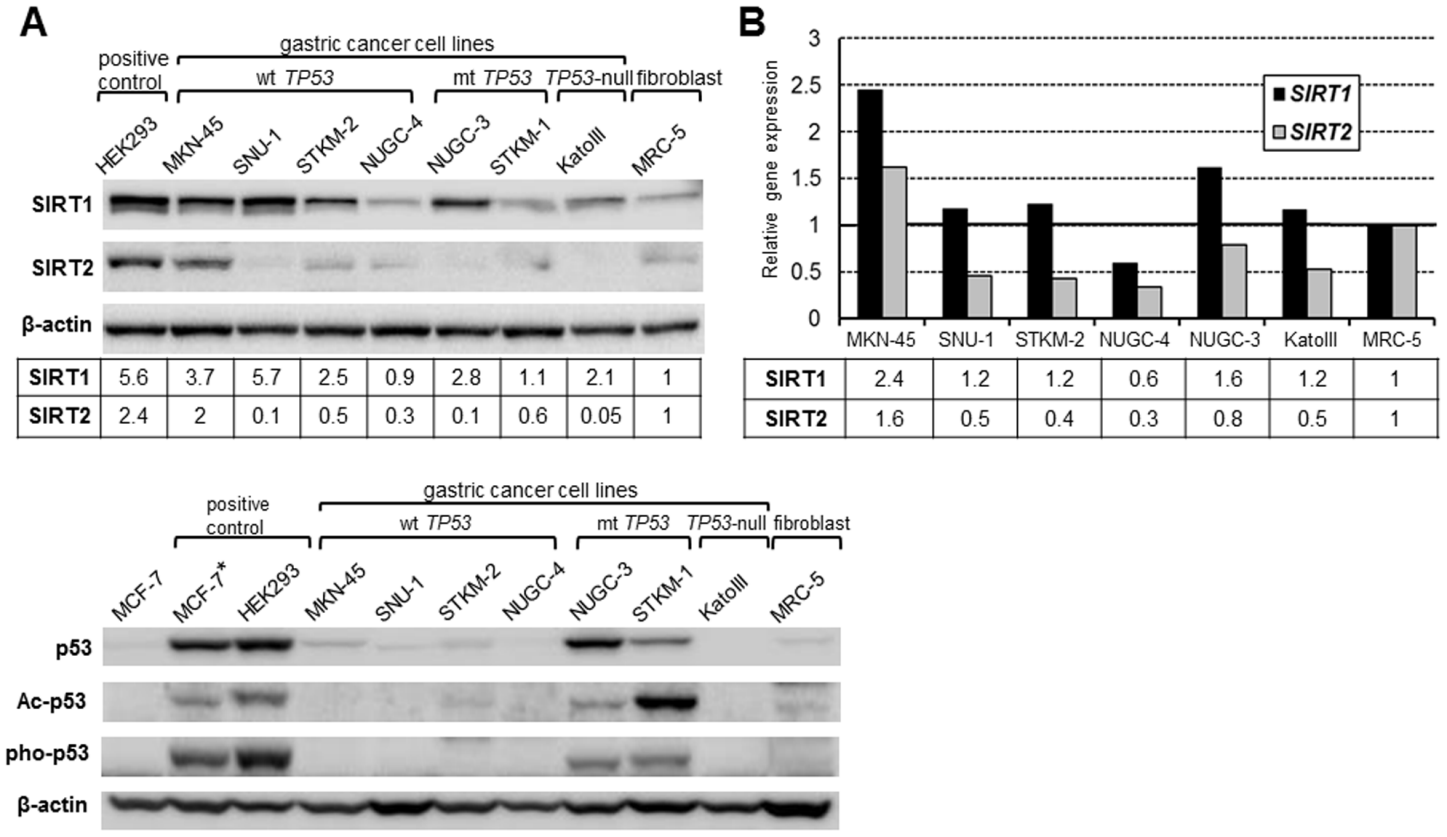
Expression of SIRT1, SIRT2, and acetylated (Ac)-p53 in gastric cancer cells. A: The expression of SIRT1, SIRT2, p53, Ac-p53, and phospho-53 in seven gastric cancer cell lines and MRC-5 fibroblasts was examined by Western blotting. Semi-quantitation of Western blotting densitometry involved normalization to β-actin levels. MCF-7*: MCF-7 cells treated with doxorubicin (1 µM, 24 h). B: Expression of *SIRT1* mRNA and *SIRT2* mRNA in gastric cancer cells. Levels of *SIRT1* mRNA and *SIRT2* mRNA were determined in gastric cancer cells and MRC-5 fibroblasts by quantitative real-time PCR and normalized to the level of *18S* rRNA. mRNA levels are shown relative to those of MRC-5 fibroblasts.

First, we examined the expression levels of SIRT1, SIRT2, and Ac-p53 in seven gastric cancer cell lines. We used HEK293 cells for the positive control of SIRT1/2, and MCF-7 cells treated with doxorubicin for the positive control of Ac-p53 [24]. All gastric cancer cell lines except for NUGC-4 and STKM-1 cells expressed high levels of SIRT1 protein, and SIRT2 expression levels were low in all cell lines except for MKN-45 cells (Figure 1A). Ac-p53 expression levels were very low in all gastric cancer cell lines with wt *TP53*.

We analyzed the gene expression of *SIRT1* and *SIRT2* by real-time quantitative PCR. MKN-45 cells exhibited *SIRT1* gene expression that was about 2.5 fold higher than that in fibroblasts, but other gastric cancer cell lines did not (Figure 1B). In NUGC-4 cells, the level of gene expression of *SIRT1* was low, as was SIRT1 protein. MKN-45 cells showed slightly high *SIRT2* gene expression, while other cell lines showed rather low expression levels.

### Tenovin-6 inhibited growth of gastric cancer cells

**Figure 2:**
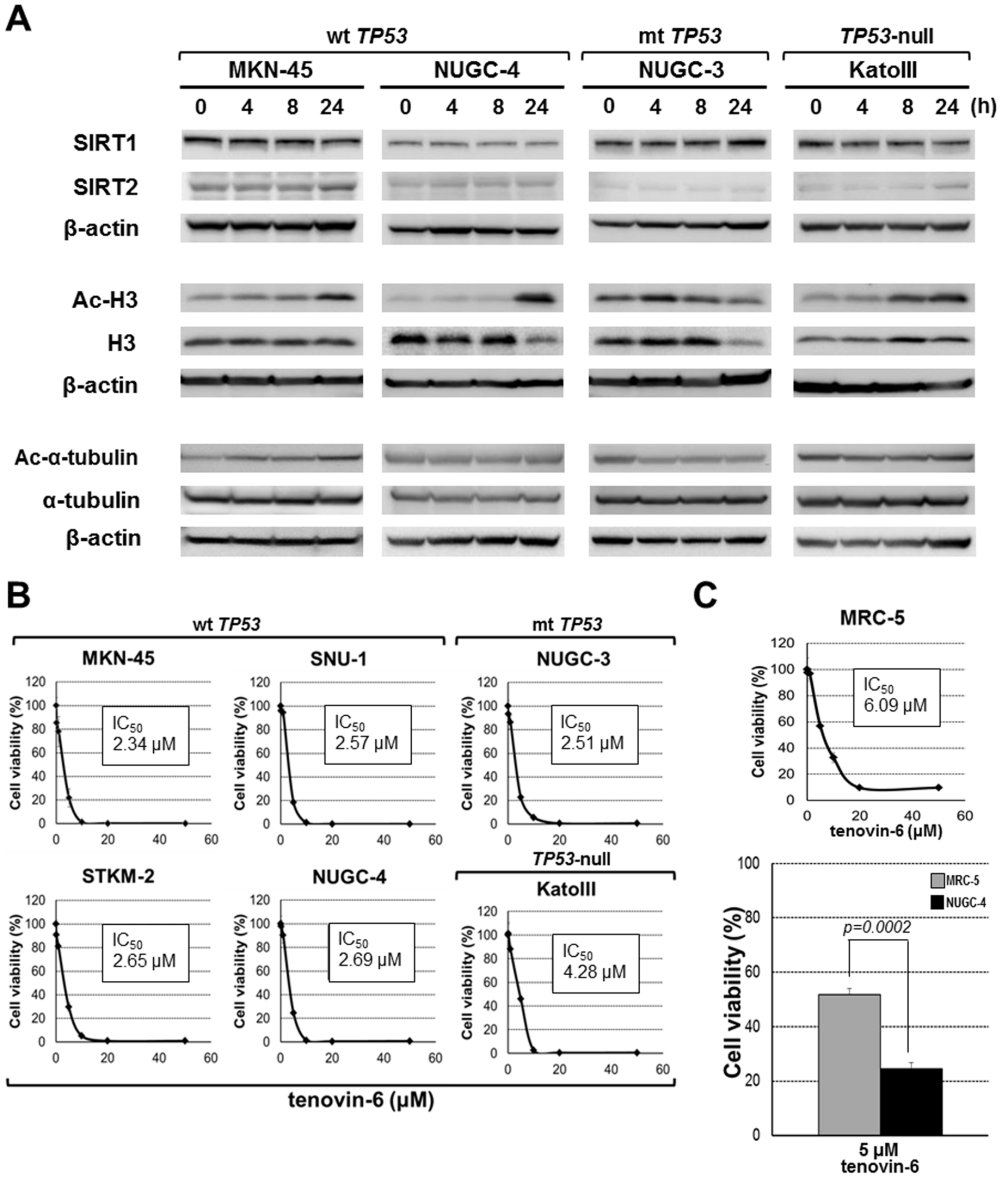
Tenovin-6-induced acetylation of H3 and α-tubulin, and antitumor effects in gastric cancer cells. A: Gastric cancer cells were cultured with tenovin-6 (10 µM) for various time periods and analyzed for the levels of SIRT1, SIRT2, Ac-H3, and Ac-α-tubulin by Western blotting. B: Tenovin-6 inhibited the growth of gastric cancer cells regardless of *TP53* status. All experiments were assessed by WST-8 assay and carried out in triplicate. Results are expressed as the mean ± SD. C: WST-8 assay was performed in MRC-5 cells to compare the toxicity of tenovin-6 to cancer cell lines. The growth inhibition of tenovin-6 in NUGC-4 cells was significantly higher than that in MRC-5 cells. The significance of differences was evaluated using Student's t-test.

In order to confirm tenovin-6 activity, we studied whether tenovin-6 affected the acetylation of histone H3 and α-tubulin. Tenovin-6 increased the acetylation of histone in three (MKN-45, NUGC-4, and KatoIII) of the four gastric cancer cell lines tested, indicating the inhibition of SIRT1 deacetylation activity (Figure 2A). Ac-α-tubulin increased only in one cell line (MKN-45) treated with tenovin-6, hence inhibition of SIRT2 deacetylation activity could not be definitely shown in gastric cancer cells.

Next, we evaluated the potential anti-tumor effect of tenovin-6 against gastric cancer cells. Each gastric cancer cell line was cultured in the presence of tenovin-6 (0.2, 1, 5, 10, 20, and 50 µM) for three days. Dose-dependent growth inhibition was observed in all cell lines, not only those with wt *TP53* but also mt and null versions (Figure 2B). Their IC_50_ values ranged from 2.34 to 4.28 µM. In addition, WST-8 assay was performed in the human fibroblast cell line MRC-5 (IC_50_: 6.09 µM) to compare the toxicity of tenovin-6 to gastric cancer cell lines. The viability of NUGC-4 cells treated with tenovin-6 was significantly lower than that of MRC-5 cells, as shown in Figure 2C.

### Tenovin-6 induced apoptotic cell death in gastric cancer cells

**Figure 3:**
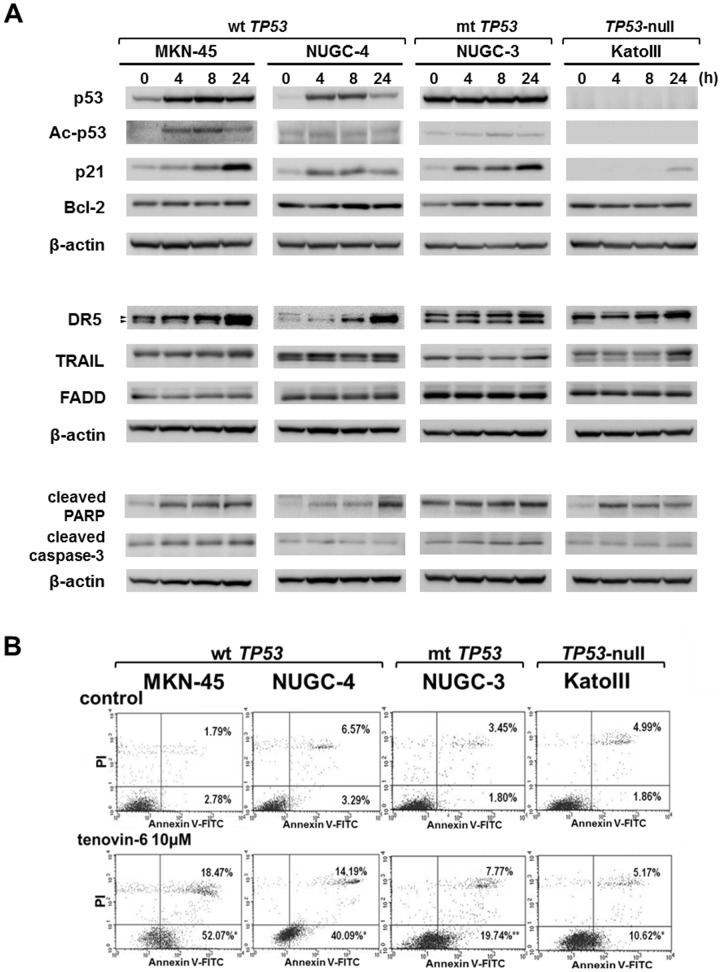
Induction of apoptotic cell death by tenovin-6 in gastric cancer cells. A: Time-course analysis of the expression of p53, its downstream molecule p21*^Waf/Cip1^*, and apoptosis-related molecules in gastric cancer cells treated with tenovin-6 (10 µM). Relative intensity of the proteins' expression is shown in Figure S1. Tenovin-6 induced the expression of Ac-p53 and p21*^Waf/Cip1^*, but not Bcl-2. DR5 expression was strongly induced by tenovin-6. TRAIL, and cleaved PARP were slightly increased, but FADD expression was not affected. A doublet of DR5 shows its precursor (upper band) and mature isoforms (lower band). B: Four cell lines were treated with tenovin-6 (10 µM) or a vehicle control for 72 h, double-stained with FITC-annexin V and PI, and analyzed by flow cytometry. The statistical significance of differences between groups was evaluated using Student's t-test. * *p*<0.01; ** *p*<0.05.

As shown in Figure 3A and S1, tenovin-6 treatment increased the expression of p53 and p21 in wt *TP53* cells (MKN45 and NUGC-4). Up-regulation of Ac-p53 was shown in MKN-45 cells, but not in NUGC-4 cells. Increased p21 expression was also observed in mt *TP53* cells (NUGC-3). By contrast, bcl-2 expression did not change in almost all four gastric cancer cell lines. Increases of DR5 and cleaved PARP expression were observed in all cell lines tested. The levels of expression of TRAIL, which function as a ligand in coupling death signaling, was slightly increased in all cell lines, although the expression of FADD, an important adaptor, was not affected by tenovin-6.

We examined whether tenovin-6 decreased the viability of gastric cancer cells through the induction of apoptotic cell death. Cancer cells were exposed to it at a concentration of 10 µM or an equivalent amount of control vehicle (DMSO) for 72 h, and then stained with FITC-annexin V and PI. They were analyzed by flow cytometry: cells negative for both annexin V and PI were considered to be non-apoptotic, cells positive for annexin V only were considered to be early apoptotic, and cells positive for both annexin V and PI were considered to be late apoptotic or necrotic. Exposure of MKN-45 cells to tenovin-6 increased the fractions of early and late phases of apoptosis from 2.8% to 52.1% and from 1.8% to 18.5%, respectively (Figure 3B). Similar increases in the populations in early and late phases of apoptosis were observed in other cell lines (NUGC-4, NUGC-3, and KatoIII). Tenovin-6 induced apoptosis in all cell lines tested, regardless of *TP53* status.

### Effect of *DR5* knockdown on tenovin-6-induced apoptosis

**Figure 4:**
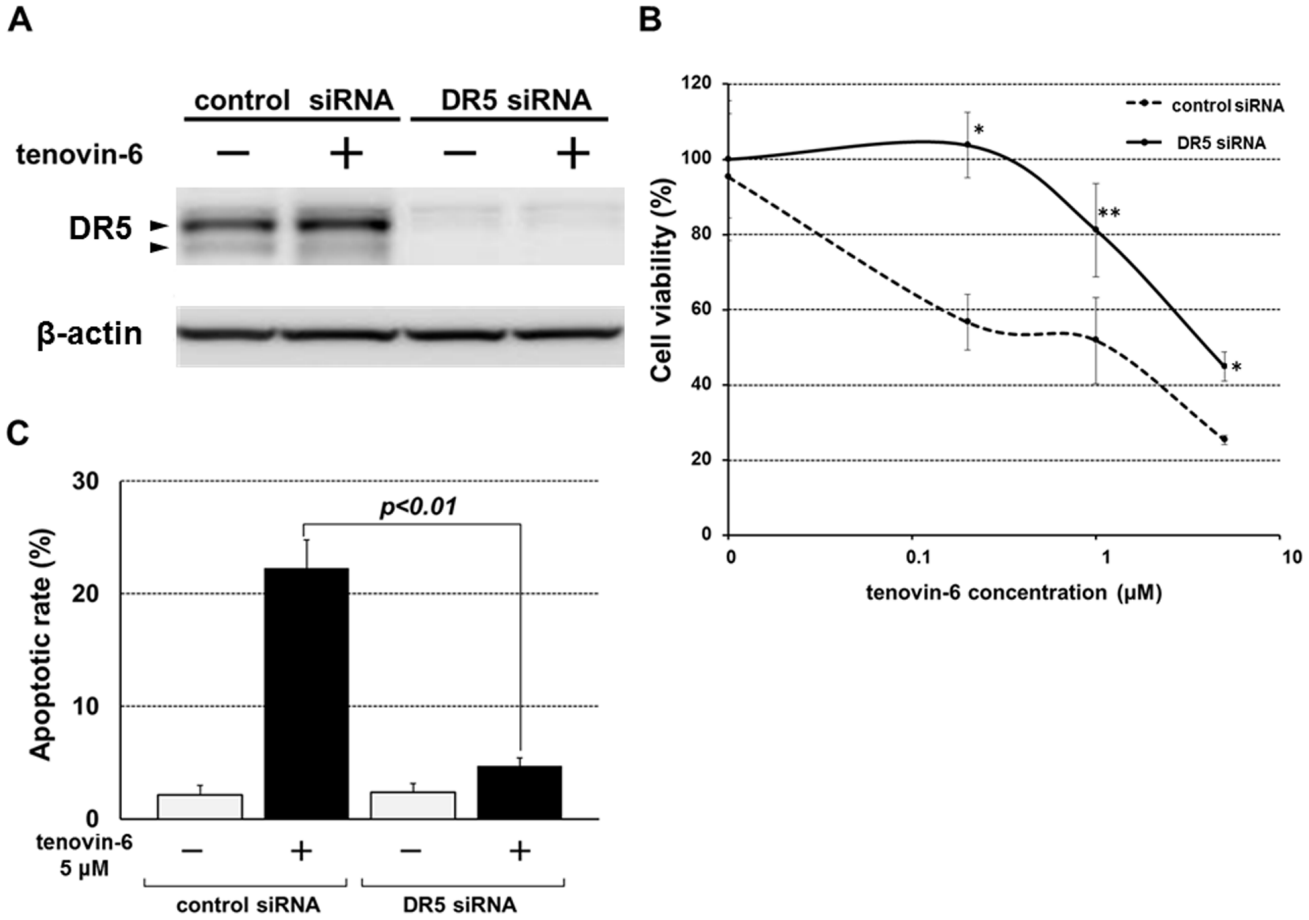
Effects of *DR5* knockdown on cell survival and apoptosis in *TP53-*null KatoIII cells. A: KatoIII cells were transfected with 1 nM siRNA control or siRNA against DR5 mRNA. Forty-eight hours after transfection, the cells were treated with 5 µM tenovin-6 for 24 h. Western blotting showed the down-regulation of DR5 by siRNA transfection. A doublet of DR5 shows its precursor (upper band) and mature isoforms (lower band). B: Cells were transfected with 1nM siRNA control or DR5 siRNA for 48 h, treated with 0.2, 1, and 5 µM tenovin-6 for 72 h and then subjected to cell viability measurements by WST-8 assay. The statistical significance of differences between groups was evaluated using Student's t-test. * *p*<0.01; ** *p*<0.05. C: The effect of DR5 knockdown on tenovin-6 induced apoptosis was analyzed by flow cytometry using PI staining. The results are expressed as the mean ± SD. The statistical significance of differences between groups was evaluated using Student's t-test.

Next, to verify whether *DR5* silencing affected tenovin-6-induced apoptosis, cell viability and apoptotic rate were analyzed by WST-8 assay and flow cytometry, respectively, in *TP53*-null KatoIII cells. Inhibition of DR5 expression by specific siRNA (Figure 4A) significantly reduced tenovin-6-induced cell death and apoptosis in KatoIII cells (Figure 4B and 4C). We used three different siRNAs in our preliminary experiment, and we got the similar results with any siRNAs.

**Figure 5:**
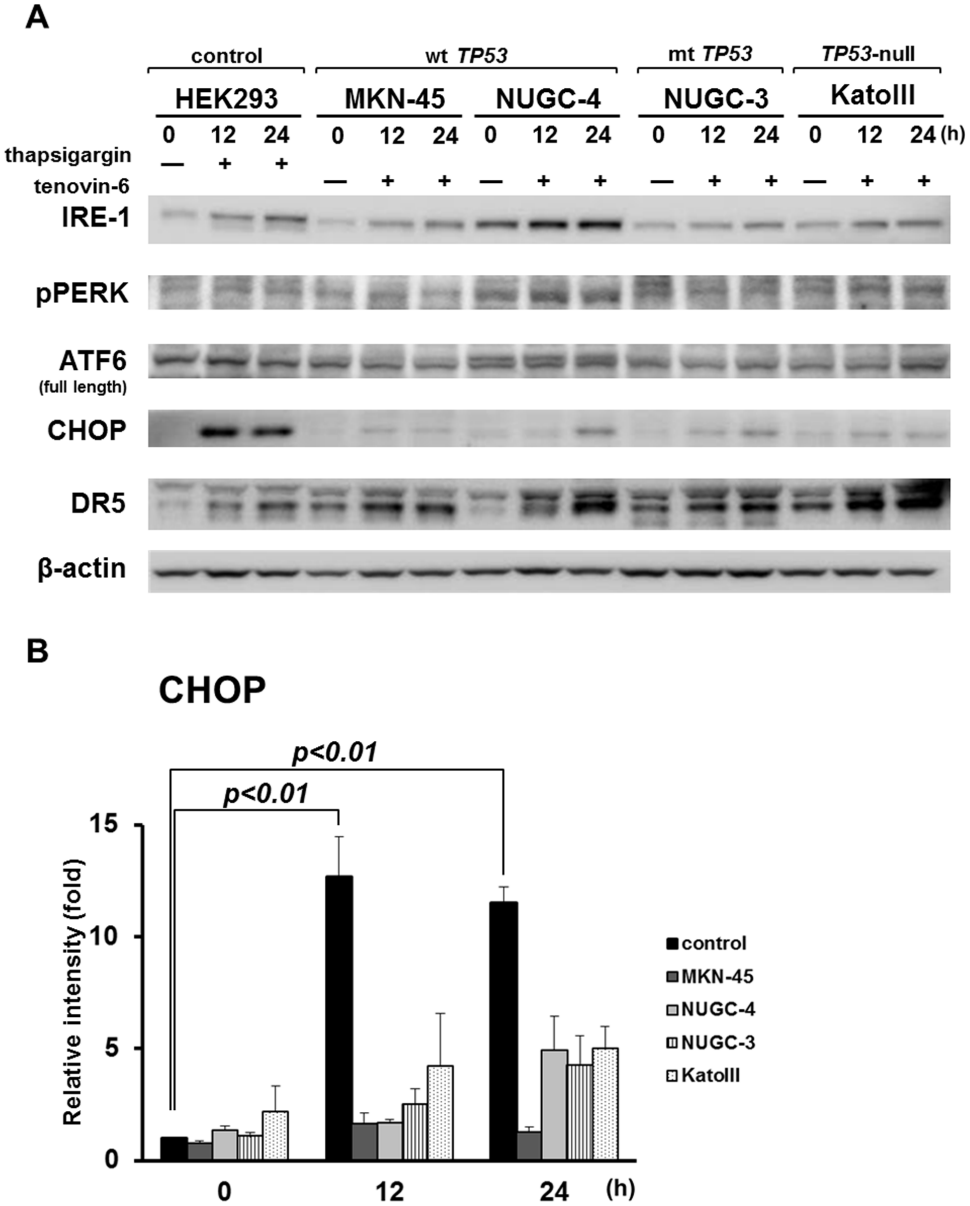
Expression of proteins associated with ER stress in gastric cancer cell lines treated with tenovin-6. A: Expression of proteins associated with ER stress in gastric cancer cell lines before and after treatment with 10 µM tenovin-6. Thapsigargin at 3 µM was administered to HEK293 cells as a control. A doublet of DR5 shows its precursor and mature isoforms. B: Relative intensity of expression of CHOP in cell lines tested is shown. The statistical significance of differences between groups was evaluated using Dunnett's test.

We investigated activation of the endoplasmic reticulum (ER) stress pathway, which is linked to DR5 up-regulation, as previously reported [17]. CHOP is one of the most potent inducer of DR5 and upstream of apoptosis, and frequently released during the ER stress. As shown in Figure 5A and 5B, although CHOP was marginally up-regulated by tenovin-6 treatment in all four gastric cancer cell lines, the increased levels were significantly lower than that in control cells treated with thapsigargin, which is a selective inhibitor of sarcoplasmic/endoplasmic reticulum Ca^2+^ - ATPases and widely used as a cellular ER stressor [25], [26]. On the other hands, IRE1, which is an ER stress sensor and located at upstream of CHOP, was slightly up-regulated by tenovin-6 treatment in all cell lines, but not phosphorylated PERK and ATF6. [27], [28]. It seemed unlikely that tenovin-6 caused ER stress leading to DR5 induction in our gastric cancer cells.

### Antitumor effect of tenovin-6 in combination with chemotherapeutic agents

Finally, we examined whether tenovin-6 enhanced the antitumor effects of chemotherapeutic agents including docetaxel, SN-38, cisplatin, and 5-FU, in gastric cancer cell lines. Four cell lines with wt *TP53* (MKN-45, NUGC-4), mt *TP53* (NUGC-3), and null *TP53* (KatoIII) were treated with these agents alone or in combination with two doses (2 and 5 µM) of tenovin-6. The concentrations were 0.25 nM docetaxel, 1 nM SN-38, 0.5 or 1 µM cisplatin, and 0.25 µM 5-FU. As shown in Table 1 (and Figure S2), docetaxel and SN-38 showed a slight to moderate synergistic effect on tenovin-6 treatment in three cell lines, and cisplatin with tenovin-6 showed a moderate synergistic effect in two cell lines, whereas 5-FU in combination with tenovin-6 showed a lower effect than the other agents. We examined the expressions of DR5 after administration of tenovin-6 with chemotherapeutic agents. DR5 up-regulation by tenovin-6 was enhanced with a combination of docetaxel in MKN-45 cells (Figure S3).

**Table 1:** Combination index of tenovin-6 plus docetaxel, SN-38, cisplatin, and 5-FU in gastric cancer cells.

tenovin-6	docetaxel	Combination index (CI)			
(µM)	(nM)	MKN-45	NUGC-4	NUGC-3	KatoIII
2	0.25	***0.867***	***0.865***	0.917	***0.847***
5	0.25	***0.890***	0.997	0.903	0.941
**tenovin-6**	**SN-38**	**Combination index (CI)**			
**(µM)**	**(nM)**	**MKN-45**	**NUGC-4**	**NUGC-3**	**KatoIII**
2	1	***0.643***	***0.804***	0.993	***0.882***
5	1	***0.785***	***0.872***	0.981	***0.895***
**tenovin-6**	**cisplatin**	**Combination index (CI)**			
**(µM)**	**(µM)**	**MKN-45**	**NUGC-4**	**NUGC-3**	**KatoIII**
2	0.5^†^, 1	***0.790***	0.966	1.020	0.919
5	0.5^†^, 1	1.079	***0.777***	0.949	0.911
**tenovin-6**	**5-FU**	**Combination index (CI)**			
**(µM)**	**(µM)**	**MKN-45**	**NUGC-4**	**NUGC-3**	**KatoIII**
2	0.25	***0.879***	1.232	1.194	0.982
5	0.25	1.232	0.945	1.141	0.928

The cytotoxic effects of combining docetaxel, SN-38, cisplatin, and 5-FU with tenovin-6 were determined by isobologram. Combination index (CI) >1.3, antagonism; CI  = 1.1–1.3 moderate antagonism; CI  = 0.9–1.1 additive effect; CI  = 0.8–0.9 slight synergism; CI  = 0.6–0.8 moderate synergism; CI  = 0.4–0.6 synergism; and CI  = 0.2–0.4 strong synergism. Bold and italic numbers indicate synergistic effects. †: NUGC-3 was treated at 0.5 µM cisplatin.

## Discussion

We demonstrated that tenovin-6 showed potent antitumor activity accompanied by apoptotic cell death in human gastric cancer cells with wt *TP53* as well as those with mt *TP53*. Several specific inhibitors of sirtuins, such as sirtinol, suramin, salermide, and thiobarbiturates, were reported to inhibit cell growth in various types of cancer [29]. Most researchers described the antitumor effects of sirtuin inhibitors in cell lines with wt *TP53* [15], [29]-[31], and attributed these to the activation of apoptosis through acetylation of p53. Meanwhile, several reports were published in recent years that showed the antitumor activities of sirtuin inhibitors in cell lines with mt *TP53* through p53-independent pathways [17], [18]. We showed that DR5 knockdown attenuated the antitumor effects of tenovin-6 in *TP53*-null gastric cancer cells. Activation of the death receptor signal pathway via up-regulation of DR5 plays a pivotal role in tenovin-6-induced cell death, as mentioned in other reports on sirtuin inhibitors.

It has been reported that salermide enhanced DR5 expression and induced apoptosis in human non-small-cell lung cancer cells carrying mt *TP53* [17]. In that report, simultaneous silencing of *SIRT1* and *SIRT2* as well as salermide up-regulated DR5, accompanied by the up-regulation of ER stress-related proteins, such as ATF4 and CHOP. These results suggested that ER stress was involved in this DR5 induction. We speculated that tenovin-6 also led to an increase of DR5 expression via activation of ER stress mediated by PERK, IRE1, ATF6, and CHOP. However, contrary to expectations, the signal pathway of ER stress activated by tenovin-6 was not obviously detected. Nevertheless, there was clear evidence that DR5 was induced by tenovin-6. There are other pathways of CHOP-mediated DR5 up-regulation: reactive oxygen species (ROS), the c-Jun NH_2_-terminal kinase (JNK) pathway, the p38 mitogen-activated protein kinase pathway, and so on [32]–[38]. However, we did not examine these pathways here because we could not identify CHOP activation in our study. Our results suggest that other p53- and CHOP-independent pathways participate in tenovin-6-induced DR5 expression in gastric cancer cells.

We demonstrated that tenovin-6 induced apoptotic cell death through activation of the DR5 pathway. However, p53 and CHOP pathways seemed unlikely to be involved in this DR5 induction, and the mechanism of DR5 up-regulation remains unclear. We have, recently, investigated antitumor effects of tenovin-6 in several colon cancer cell lines, and found its potent antitumor activity against most of them with up-regulation of DR5 as well [39]. However in CaCo2 colon cancer cells (mt *TP53*), apoptotic cell death by tenovin-6 was less evident and DR5 expression was not strongly up-regulated. CaCo2 cell have been reported to express high level of heat shock proteins known as a suppressor of DR5 [40]-[43]. This relation should be studied further in future. SIRT1 can deacetylate histone H4 lysine 16 (H4K16) as well as H3K9, H3K14, and H1K26, which are closely related to gene silencing [11]. In addition, it has many corresponding non-histone substrates: transcriptional factors, DNA repair machinery elements, nuclear receptors, histone-modifying enzymes, and cell signaling molecules, as described elsewhere [11]–[13]. These numerous and complicated associations of SIRT1 activity are involved in various biological functions, such as regulation of gene expression and DNA damage repair. Cancer cells tend to require these functions of SIRT1 in order to survive, proliferate, and repair catastrophic genomic damage. Recent studies have identified the ability of tenovin-6 to induce differentiation and inhibit autophagy as part of its anti-neoplastic effects in leukemia cells [44], [45]. It may depend on cancer cell behavior how tenovin-6 affects neoplastic activity. Further studies are needed to clarify the link between the tenovin-6-mediated death pathway and the complex roles of SIRT1.

We examined the effects of combining docetaxel, SN-38, cisplatin, and 5-FU with tenovin-6 because they have been widely used for the treatment of patients with advanced gastric cancer [46], [47]. Slight to moderate synergistic effects of docetaxel and SN-38 with tenovin-6 in the gastric cancer cell lines, regardless of *TP53* status, were found.

Although treatments involving combinations of docetaxel and sirtuin inhibitors have not been reported, combinations of docetaxel and other HDAC inhibitors such as trichostatin A and suberoylanilide hydroxamic acid (SAHA) have been reported to have synergistic effects related to caspase activation or tubulin acetylation in several cancer cell lines [48]–[50]. In our study, it remains unclear if the tubulin acetylation by tenovin-6 always affected the antitumor effect, because the tubulin acetylation was shown only in one cell line. SN-38 (the active form of irinotecan) is a DNA topoisomerase I inhibitor that acts only during the S phase and interferes with DNA replication and cell division [51], [52]. HDAC inhibitors induce acetylation of histones and loosen the chromatin structure, whereby topoisomerase inhibitor may more easily access DNA, facilitating DNA damage [51], [53]. In addition, a remarkable increase of ROS generation has been reported in small-cell lung cancer cells upon simultaneous exposure to SAHA and topotecan (a derivative of camptothecin) [51]. The increased potency of treatment combining tenovin-6 and SN-38 might be attributable to cooperative regulation of the DNA damage response.

The advantage of combined therapy with tenovin-6 and cisplatin or 5-FU was less than that with the other agents in gastric cancer cells, although several reports have demonstrated the enhancement of apoptosis by combined treatment with cisplatin or 5-FU and other HDAC inhibitors in other tumors [54]–[57].

Regarding the toxicity evaluation of tenovin-6, we used a fibroblast cell line as the alternative non-tumorigenic cells to predict the toxicity against normal cells referring to the previous reports [15], [18]. It was difficult to find an appropriate normal control for comparative studies, and is therefore definitively needed to study the toxicity of tenovin-6 (in combination with anti-cancer drugs) *in vivo*, using animal xenograft models.

In conclusion, a sirtuin inhibitor, tenovin-6, showed a robust antitumor effect against human gastric cancer cells. This was independent of *TP53* status and was induced via up-regulation of DR5. Further study is needed to clarify the mechanism by which tenovin-6 regulates DR5 expression. Tenovin-6 combined with docetaxel and SN-38 had a small advantage for inhibition of gastric cancer cell proliferation, which could provide a novel strategy for the treatment of advanced gastric cancer.

## Supporting Information

Figure S1 **Relative intensity of the proteins' expression shown in** **Figure 3A** **.** Semi-quantitation of Western blotting densitometry involved normalization to β-actin levels.
(TIF)

Figure S2 **Cytotoxic effects of chemotherapeutic drugs in combination with tenovin-6.** Cytotoxic effects of chemotherapeutic drugs including docetaxel, SN-38, cisplatin, and 5-FU, and their enhancement of the effects of tenovin-6 in gastric cancer cells with gastric cancer cells. The cells were cultured for 72 h with the indicated concentrations of tenovin-6 and chemotherapeutic drugs. A: Docetaxel (0.25 nM), B: SN-38 (1 nM), C: cisplatin (1 or 0.5 µM; NUGC-3 was treated with 0.5 µM cisplatin), and D: 5-FU (0.25 µM) were given in combination with tenovin-6. The statistical significance of differences between groups was evaluated using Dunnett's test. * *p*<0.01; ** *p*<0.05. DOC: docetaxel, CDDP: cisplatin.
(TIF)

Figure S3 **DR5 expressions after administration of tenovin-6 with docetaxel or SN-38 in gastric cancer cells (MKN-45 and KatoIII).** Docetaxel, SN-38 and tenovin-6 were administrated at a concentration of 0.25 nM, 1 nM and 2 µM. Semi-quantitation of Western blotting densitometry involved normalization to β-actin levels. DOC: docetaxel.
(TIF)
