# Supplementary figures and images for: Antitumor Effects of a Sirtuin Inhibitor, Tenovin-6, against Gastric Cancer Cells via Death Receptor 5 Up-Regulation
Source: PLoS One. 2014 Jul 17;9(7):e102831. doi: 10.1371/journal.pone.0102831 (PMC4102575; doi:10.1371/journal.pone.0102831)

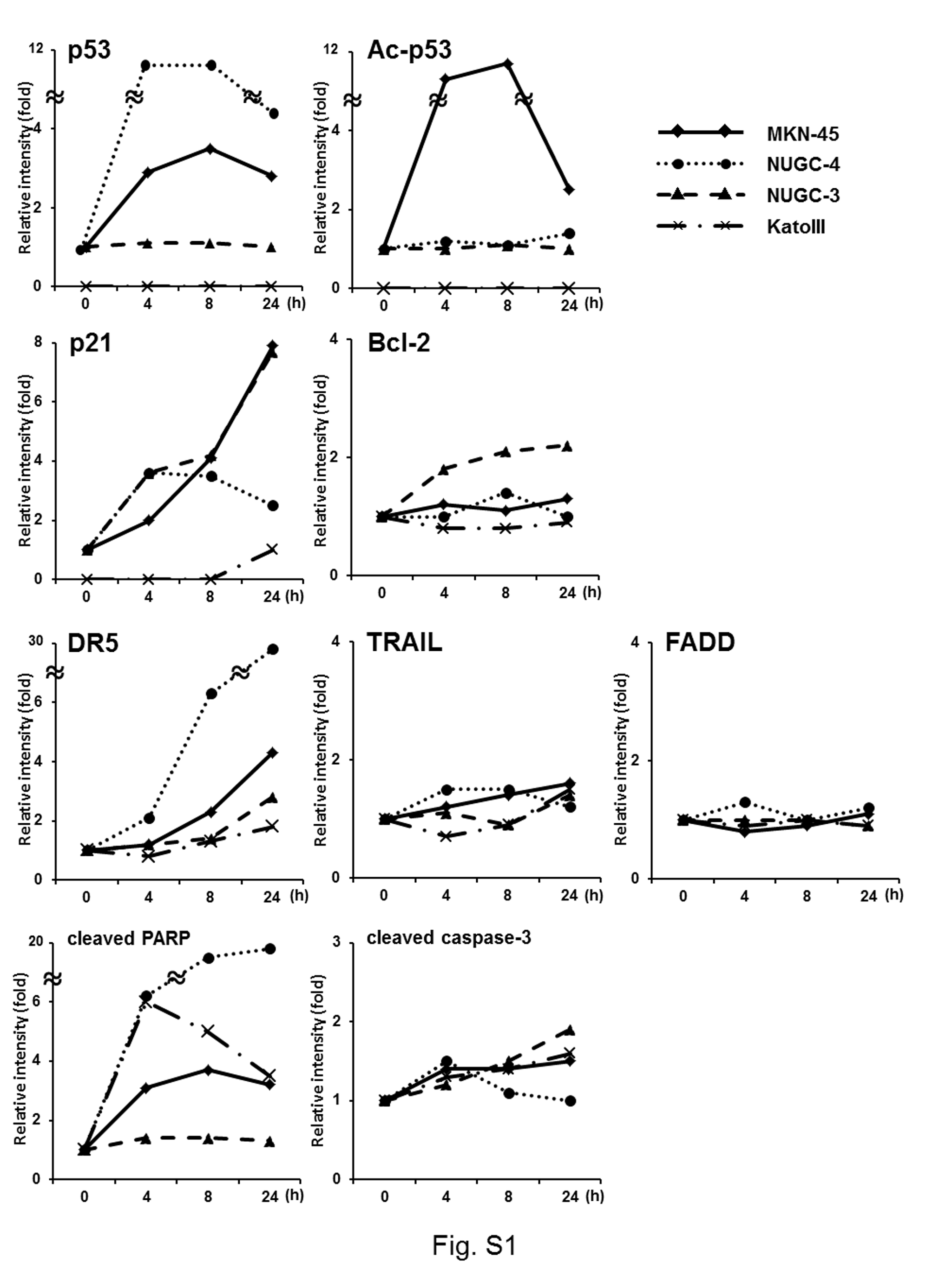

Supplement: Figure S1 — Relative intensity of the proteins' expression shown in Figure 3A . Semi-quantitation of Western blotting densitometry involved normalization to β-actin levels. (TIF) [file pone.0102831.s001.tif]

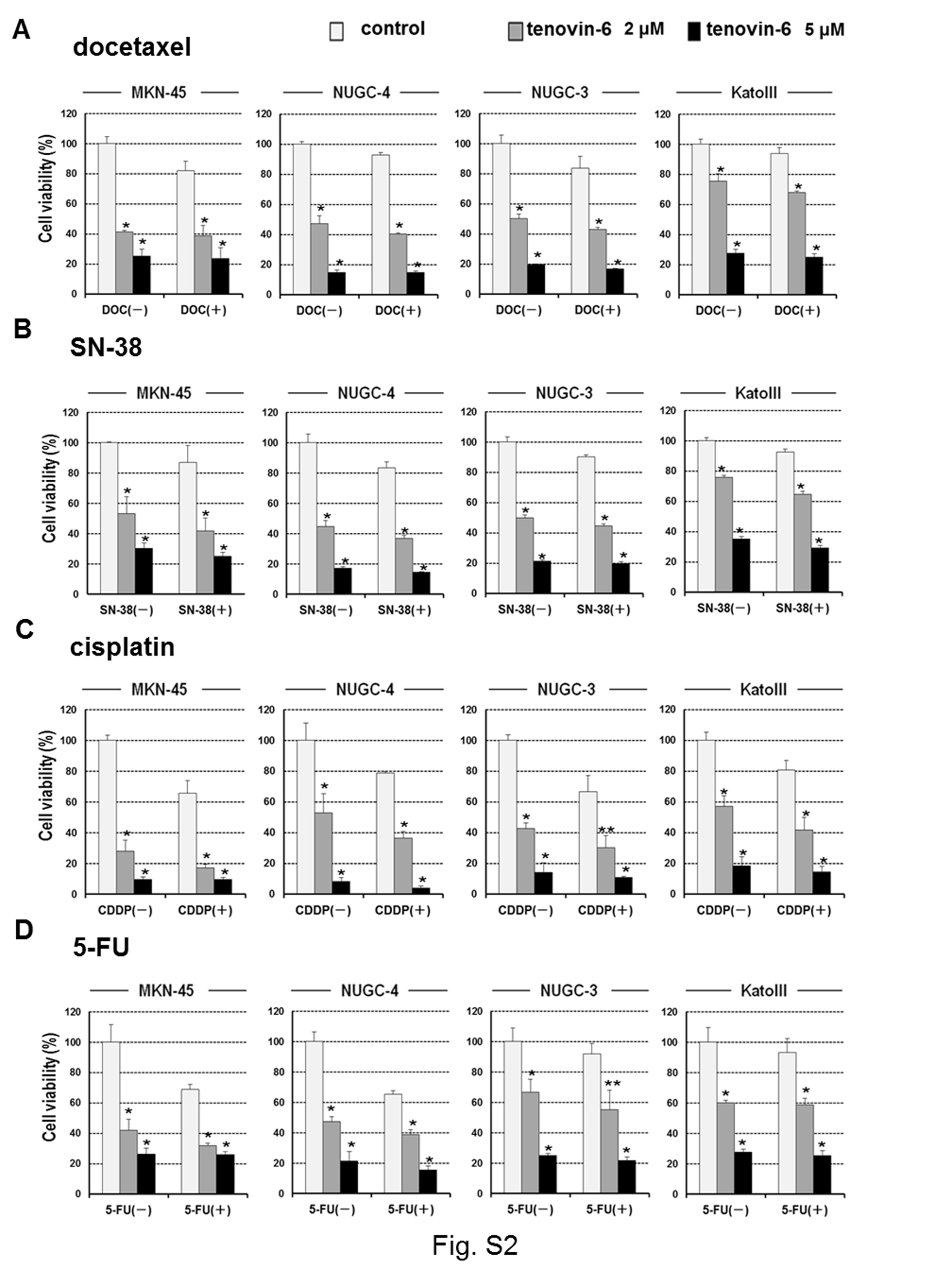

Supplement: Figure S2 — Cytotoxic effects of chemotherapeutic drugs in combination with tenovin-6. Cytotoxic effects of chemotherapeutic drugs including docetaxel, SN-38, cisplatin, and 5-FU, and their enhancement of the effects of tenovin-6 in gastric cancer cells with gastric cancer cells. The cells were cultured for 72 h with the indicated concentrations of tenovin-6 and chemotherapeutic drugs. A: Docetaxel (0.25 nM), B: SN-38 (1 nM), C: cisplatin (1 or 0.5 µM; NUGC-3 was treated with 0.5 µM cisplatin), and D: 5-FU (0.25 µM) were given in combination with tenovin-6. The statistical significance of differences between groups was evaluated using Dunnett's test. * p<0.01; ** p<0.05. DOC: docetaxel, CDDP: cisplatin. (TIF) [file pone.0102831.s002.tif]

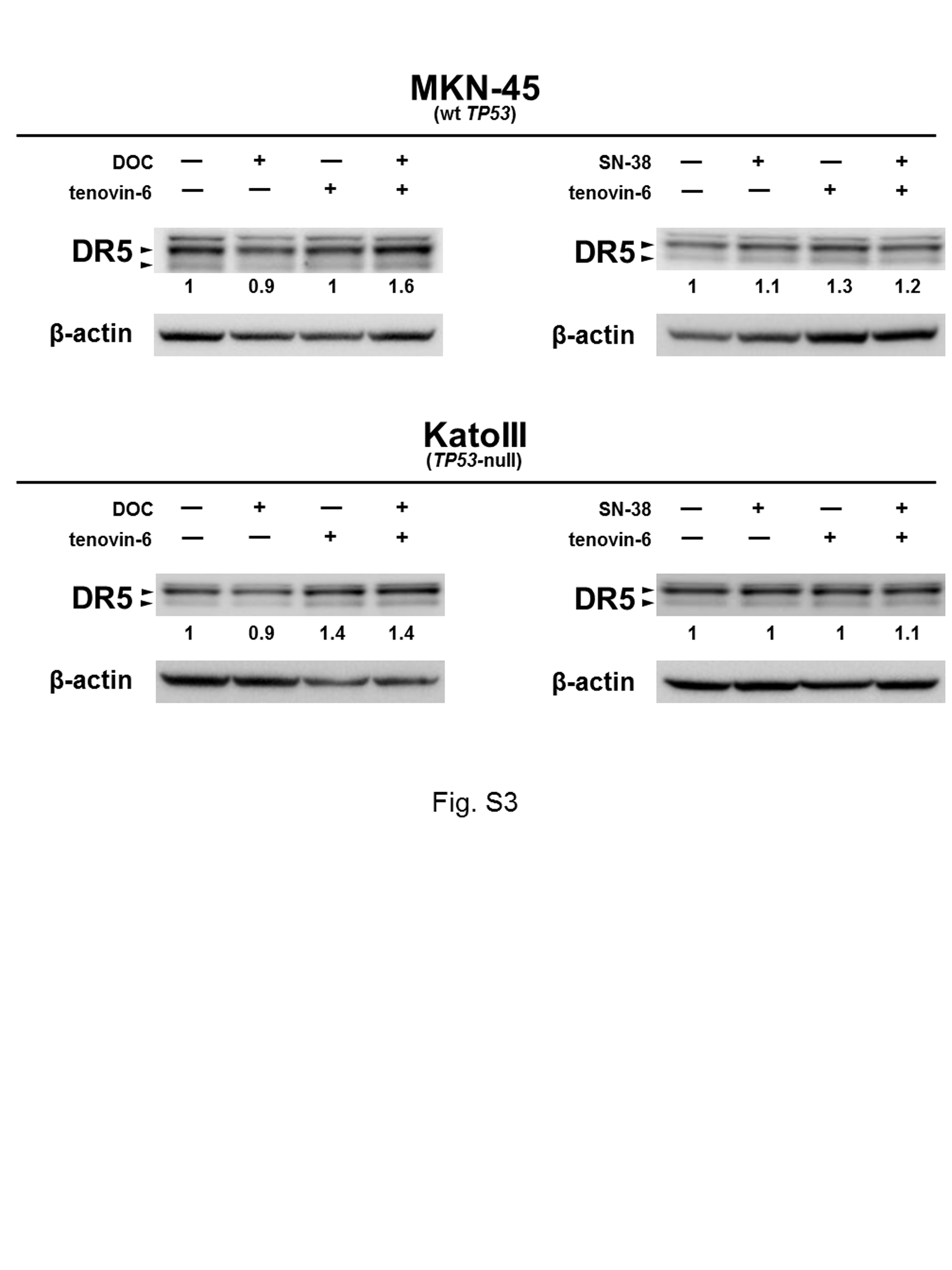

Supplement: Figure S3 — DR5 expressions after administration of tenovin-6 with docetaxel or SN-38 in gastric cancer cells (MKN-45 and KatoIII). Docetaxel, SN-38 and tenovin-6 were administrated at a concentration of 0.25 nM, 1 nM and 2 µM. Semi-quantitation of Western blotting densitometry involved normalization to β-actin levels. DOC: docetaxel. (TIF) [file pone.0102831.s003.tif]
